# Synthesis and Characterization of ABA-Type Triblock Copolymers Using Novel Bifunctional PS, PMMA, and PCL Macroinitiators Bearing *p*-xylene-bis(2-mercaptoethyloxy) Core

**DOI:** 10.3390/polym15183813

**Published:** 2023-09-18

**Authors:** Murat Mısır, Sevil Savaskan Yılmaz, Ahmet Bilgin

**Affiliations:** 1 Faculty of Engineering and Architecture, Department of Chemical Engineering, Kırşehir Ahi Evran University, Kırşehir 40100, Turkey; murat.misir@ahievran.edu.tr; 2 Faculty of Sciences, Department of Chemistry, Karadeniz Technical University, University Avenue, Trabzon 61080, Turkey; 3 UNAM–National Nanotechnology Research Center and Institute of Materials Science and Nanotechnology, Bilkent University, Ankara 06800, Turkey; 4 Faculty of Education, Department of Mathematics and Science Education, Kocaeli University, Kocaeli 41001, Turkey; abilgin@kocaeli.edu.tr

**Keywords:** the novel bi-functional ATRP and ROP initiator, ring opening polymerization, atom transfer radical polymerization, ABA–type triblock copolymer, heavy metal extraction

## Abstract

Syntheses of novel bifunctional poly(methyl methacrylate) (PMMA)-, poly(styrene) (PS)-, and (poly *ε*-caprolactone) (PCL)-based atom transfer radical polymerization (ATRP) macroinitiators derived from *p*-xylene-bis(1-hydroxy-3-thia-propanoloxy) core were carried out to obtain ABA-type block copolymers. Firstly, a novel bifunctional ATRP initiator, 1,4-phenylenebis(methylene-thioethane-2,1-diyl)bis(2-bromo-2-methylpropanoat) (PXTBR), synthesized the reaction of *p*-xylene-bis(1-hydroxy-3-thia-propane) (PXTOH) with α-bromoisobutryl bromide. The PMMA and PS macroinitiators were prepared by ATRP of methyl methacrylate (MMA) and styrene (S) as monomers using (PXTBR) as the initiator and copper(I) bromide/*N*,*N*,*N′*,*N″*,*N″*-pentamethyldiethylenetriamine (CuBr/PMDETA) as a catalyst system. Secondly, di(α-bromoester) end-functionalized PCL–based ATRP macronitiator (PXTPCLBr) was prepared by esterification of hydroxyl end groups of PCL-diol (PXTPCLOH) synthesized by Sn(Oct)_2_–catalyzed ring opening polymerization (ROP) of *ε*-CL in bulk using (PXTOH) as initiator. Finally, ABA-type block copolymers, PXT(PS-*b*-PMMA-*b*-PS), PXT(PMMA-*b*-PS-*b*-PMMA), PXT(PS-*b*-PCL-*b*-PS), and PXT(PMMA-*b*-PCL-*b*-PMMA), were synthesized by ATRP of MMA and S as monomers using PMMA-, PS-, and PCL-based macroinitiators in the presence of CuBr/PMDETA as the catalyst system in toluene or *N*,*N*-dimethylformamide (DMF) at different temperatures. In addition, the extraction abilities of PCL and PS were investigated under liquid–liquid phase conditions using heavy metal picrates (Ag^+^, Cd^2+^, Cu^2+^, Hg^2+^, Pb^2+^, and Zn^2+^) as substrates and measuring with UV-Vis the amounts of picrate in the 1,2–dichloroethane phase before and after treatment with the polymers. The extraction affinity of PXTPCL and PXTPS for Hg^2+^ was found to be highest in the liquid–liquid phase extraction experiments. Characterizations of the molecular structures for synthesized novel initiators, macroinitiators, and the block copolymers were made by spectroscopic (FT–IR, ESI–MS, ^1^H NMR, ^13^C NMR), DSC, TGA, chromatographic (GPC), and morphologic SEM.

## 1. Introduction

Among controlled/living radical polymerization (CRP) techniques, ATRP, a highly interesting method based on a copper halide/nitrogen-based ligand catalyst system, has found great usage for macromolecular design using halogen-containing initiators [1,2,3,4,5,6]. The ATRP method, known for regulating chain length and functionality [7], has been demonstrated for the controlled polymerization of different monomers, which include a variety of functional groups, such as styrene [8,9,10], acrylates [11,12], and methacrylates [13,14]. Additionally, this method provides control for variations in composition and architecture [15]. Moreover, ATRP has a considerable attraction for preparing definite polymers that have special functionalities since it shows tolerance against functional groups and impurities [8,16,17]. The ATRP technique has a special initiating system consisting of three components. These components are mainly halide-type initiators (alkyl halide), catalysts (a transition metal salt in the lower oxidation state), and a complexing ligand (based on amine–type or organophosphorus compounds) [16,17,18]. Among them, initiator efficiency is crucial for performing a successful ATRP reaction because it determines the number of initiated chains [19]. The use of functional initiators and monomers with the chemical transformation of the halogen end groups in ATRP is important with respect to polymer functionalization [20]. ATRP initiators mostly contain either bromine or chlorine as the halogen group in the initiating stage of polymerization. The structure of the alkyl group and transferable (pseudo)-halogen determines the reactivities of alkyl halides (RX) in ATRP [3].

Over the last decades, the synthesis of block copolymers (BCPs) by controlled polymerization techniques such as ATRP, reversible addition-fragmentation chain transfer (RAFT) radical polymerization, nitroxide-mediated polymerization (NMP), and some others has been described in numerous publications. BCPs can be prepared using the ATRP technique by adding a second monomer to polymer chain ends equipped with an alkyl halide [21].

Macromonomeric initiators act as macromonomers, macroinitiators, or macrocrosslinkers and have attracted substantial interest due to them being able to synthesize crosslinked or branched block copolymers [22,23,24]. In order to ensure successful ATRP, initiators need to be equipped with enhanced radical stabilization by the presence of more acting groups on the carbon atom, such as aryl, carbonyl, nitrile, and multiple halogens, in addition to desired functional groups [25].

The advancements in synthetic methods have enabled the synthesis of various kinds of di and triblock copolymers. Using many polymerization routes, the preparation of AB-type diblock or ABA-type triblock copolymers containing both PS and PMMA has been extensively studied by many researchers. AB-type PMMA-*b*-PS BCPs were prepared and studied by some researchers using polymerization methods. Huang et al. [26] synthesized PMMA-*b*-PS by two consecutive ATRPs. Aimi et al. [27] prepared phthalocyanine (Pc)-tethered (Pc-PMMA-*b*-PS) using a click reaction between the alkynylated Pc and azide-functionalized PMMA-*b*-PS block copolymer synthesized sequentially with ATRP. Dithiobenzoate-terminated PMMA-*b*-PS was prepared using a PMMA macroinitiator via two consecutive RAFTs by Spruell et al. [28]. PMMA-*b*-PS grafted onto the surface of TiO_2_ by RAFT polymerization was studied by Xiong et al. [29]. There are also several research papers for AB–type PS-*b*-PMMA BCP in the literature. For example, Lee et al. [30] synthesized PS-*b*-PMMA diblock copolymers via a click reaction using the PS with an alkyne end group by ATRP and azide-functionalized PMMA by anionic polymerization, respectively. Brittain et al. [31] studied the synthesis of PS-*b*-PMMA block copolymer films on a silicate substrate by sequential carbocationic polymerization of S followed by ATRP of MMA. Khaydarov et al. [32] studied a synthetic approach to asymmetric PS-*b*-PMMA diblock copolymer BPCs synthesized via RAFT. Bolton et al. [33] followed a synthetic approach to PS-*b*-PMMA bottlebrush block copolymer using a combination of ATRP and RAFT consecutively. Temel et al. [34] showed that PS-*b*-PMMA was prepared by a photo-induced radical coupling process using end-chain benzophenone-functionalized PS and PMMA obtained by ATRP. Aitchison et al. [35] prepared PS-*b*-PMMA by sequential ARGET-ATRP.

The synthesis of the ABA-type triblock copolymer (TBC) can be accomplished by sequential monomer addition, synthesis of the AB-type diblock, and subsequent coupling reaction, as well as by sequential polymerization of monomers initiating from a bi-functional initiator [21]. Bifunctional initiators are attractive components in polymerization because they have the same reactivity at both chain ends. Therefore, these can induce bidirectional chain growth in the reaction [36]. This property of these initiators allows them to prepare telechelic polymers, and diblock or triblock copolymers [36,37,38].

PMMA and PS macroinitiators synthesized via CRP methods using difunctional [39,40,41,42] initiators have been employed for the preparation of ABA-type BCPs. Not only AB-type diblock but also ABA-type triblock copolymers could be synthesized by sequential addition regardless of monomers, S, or MMA. Hirao et al. [43] reported that PS-*b*-PMMA-*b*-PS was synthesized by living anionic polymerization sequential polymerization adding monomers to anionic initiators using a phenylacrylate linking reaction site. Karkare et al. [44] synthesized PMMA-*b*-PS-*b*-PMMA by combining both ATRP and ARGET-ATRP using -cyclodextrin (-CD). Wu et al. [45] used PMMA-*b*-PS-*b*-PMMA triblock copolymers for the preparation of long multi–block copolymer chains of the related copolymer via the self-assembly assisted polycondensation (SAAP) method. Toman et al. [46] synthesized PS-*b*-PMMA-*b*-PS via consecutive ATRPs using a difunctional initiator bearing trichloromethyl groups.

Telechelic polymers defined as polymeric molecules with reactive end groups of the capacity to enter into further polymerization or other modifications [37] can generally be classified as homobifunctional and heterobifunctional telechelic polymers. Homobifunctional telechelics, prepared by the use of a bifunctional initiator after polymerization and termination or chain end modification, have the same functional group at both chain ends, while the heterobifunctional telechelics of the functional group at the R-chain end differ in that they are present at the -chain end [47]. Depending on their functionality, telechelics can also be classified as mono- or semi-, di-, tri-, and multifunctional telechelics (polytelechelics) [37]. The use of a well–defined initiator in controlled polymerizations provides control over molecular weight and polydispersity. End-functional homopolymers are important in the synthesis of multiblock copolymers, graft copolymers, star–shaped architectures, and cross-linked networks [47]. Post-polymerization chemical modification of chain-end polyester diols into other functional group(s) was next used as a macroinitiator to prepare copolymers [48]. Among the different types of multiblock copolymers, one of the most common techniques is sequential addition of the monomer(s), which was successfully used to synthesize polymers by using the monofunctional initiating system to form AB, ABC, and ABA types or by using the bifunctional initiator to form BAB and CBABC types [49].

Among controlled radical polymerizations (CRP), the combination of ROP and ATRP has received remarkable attention with regard to macroinitiator and block copolymer synthesis [50]. ROP can be performed both in a controlled and living manner, depending on the monomer and initiator/catalyst system [51]. Aliphatic polyesters such as poly (L-lactic acid) (PLLA) and poly(*ε*–caprolactone) (PCL) are interesting polymers for pharmacological, biomedical, environmental, and agricultural applications due to their good mechanical properties, hydrolyzability, and biocompatibility [52,53,54]. Recently, copolymers and blends of these homopolymers have been studied to enhance their mechanical, thermal, or biodegradable properties.

PCL, approved by the FDA, is a commercially attractive, biodegradable polymer used in drug delivery systems or as a synthetic biomaterial because of its predictable biodegradability, high drug permeability, and biocompatibility [55].

The slow degradation of PCL and the absence of an acidic environment during the degradation process make this polymer suitable for long–term drug release [56]. By careful selection of the initiator systems, PCLs functionalized with different end groups have been obtained, and these groups provide a wide range of possibilities for the synthesis of PCL-based copolymers with advanced structures such as block, star-shaped, comb-like, brush-like, and cross-linked network structures. PCL-based block copolymers are also interesting because of various factors such as their miscibility with a wide range of polymers, crystallizability, lack of toxicity, printability, and low-temperature adhesiveness. They are also used as dispersants (for pigments), surfactants, compatibilizers, etc.

Many research groups have studied PCL-based block copolymers with amphiphilic and double hydrophobic characters via the combination of various CRP methods along with ROP. Generally, it is possible to obtain PCL-based block copolymers using polymerization reactions such as ATRP, NMP, RAFT, and click conjugation with ROP. The reported studies on the synthesis of PCL and PMMA-based block copolymers have generally been carried out by following different routes. Recent efforts have demonstrated that AB- and ABA-type block copolymers based on PCL with PMMA or PS could be prepared by a combination of ROP and ATRP using dual or symmetric bifunctional initiators. For example, Yilmaz [57] reported a one–pot synthetic approach to BCPs via a combination of photo-induced metal-free ATRP and ROP processes using difunctional initiators for the preparation of AB2-type star PS-*b*-(PCL)_2_ and PMMA-*b*-(PCL)_2_ BCPs. Schappacher et al. synthesized AB- and ABA-type BCPs based on PMMA and PCL by combining ROP and ATRP [58]. Yuan et al. prepared star-shaped PCL-*b*-PS and PCL-*b*-PMMA BCPs via a combination of ATRP and ROP initiated by hydroxyl-terminated cyclotriphosphazane [59]. PCL-*b*-PS star BCPs were synthesized by the combination of lipase-catalyzed ROP and ATRP [60]. A2B2-type PCL-*b*-PS miktoarm polymers with pentaerythritol cores by combination of ROP and ATRP [61] A3B3–type PCL-*b*-PMMA miktoarm star polymers were reported by sequential polymerization on a six-armed dendrimer with alternating free hydroxyl and bromobutyrate end groups by ROP and ATRP, respectively [62]. Combining enzymatic ROP and ATRP produced an ABA2-type (Y-shaped) BCP made up of PCL and PS blocks [63]. Preparation of ABA-type PDMAEMA-*b*-PCL-*b*-PDMAEMA BCP initiated by difunctional PCL macroinitiators was successful [64]. Huang et al. reported ABC–type BCPs were prepared through ROP and two specific ATRPs, *n*-butyl acrylate and methyl methacrylate [65]. AB-type (PCL)-*b*-(polyvinyl acetate) (PVAc) BCP was investigated using click chemistry, RAFT, and ROP techniques [66].

In this work, the novel TBCs consisting of PS and PMMA were synthesized by two different pathways using novel ATRP (PXTBr) and ROP (PXTOH) initiators. We chose the sulfur-containing compound as a novel initiator because sulfur-containing initiators have been relatively less commonly used than those containing oxygen and nitrogen. Moreover, sulfur-containing compounds can degrade at high temperatures, making them difficult to use in ATRP. In addition to the disadvantages mentioned above, we aimed to observe how polymerization proceeds with sulfur–containing initiators, while low efficiency was expected because copper(I) has a soft acid character and sulfur has a soft base character, allowing easy coordination with them. The negative effects of heavy metals in wastewater have been a known issue for a long time since they have hazardous effects on the environment and human health. Thus, it could be logical to use sulfur-containing polymers to remove these metals from wastewater [67].

Firstly, the novel bifunctional ATRP initiator (PXTBr) was synthesized and employed for polymerization of MMA and S using CuBr/PMDETA as a catalyst system in bulk. In addition to these macroinitiators, di(-bromoester) end-functionalized PXTPCLBr was prepared by esterification of hydroxyl end groups of PXTPCLOH synthesized by Sn(Oct)_2_–catalyzed ROP of *ε*-CL in bulk using the difunctional initiator. ABA-type block copolymers were synthesized by second ATRP of MMA and S as monomers using novel (PMMA-, PS-, and PCL-based) macroinitiators and CuBr/PMDETA as catalyst systems in toluene or DMF at different temperatures. In the final step, the heavy metal extraction abilities of synthesized polymers (PXTPCL and PXTPS) were also investigated under liquid–liquid phase conditions using heavy metal picrates (Ag^+^, Cd^2+^, Cu^2+^, Hg^2+^, Pb^2+^, and Zn^2+^) as substrates and measuring with UV-Vis the amounts of picrate in the 1,2-dichloroethane phase before and after treatment with the polymers. The extraction affinity of polymers for Hg^2+^ was found to be highest in the liquid–liquid phase extraction experiments.

The synthesized novel initiators were characterized by spectroscopic methods (FT–IR, ESI–MS, ^1^H NMR, and ^13^C NMR). The obtained macroinitiators and BCPs were also characterized by DSC, TGA, GPC, and SEM.

## 2. Experimental Section

### 2.1. Materials

Reactions were performed under an atmosphere of nitrogen using standard Schlenk techniques unless otherwise specified. MMA (Aldrich, St. Louis, MO, USA, 99%) and S (Aldrich, St. Louis, MO, USA, 99%) were washed with a 5 wt% aqueous NaOH (Aldrich, St. Louis, MO, USA) solution five times to remove inhibitors and water until the aqueous phase became neutral, then dried and vacuum–distilled before use. The monomers were deoxygenated by purging with dry nitrogen gas for 2 h prior to being stored at 0 °C. *ε*-CL (ABCR GmbH & Co. KG, Karlsruhe, Germany, 99%) was purified by distilling CaH_2_. Tetrahydrofuran (THF, Aldrich, St. Louis, MO, USA, 99%) was distilled from sodium and benzophenone under an argon atmosphere. Toluene was distilled from sodium and benzophenone. DMF was distilled from magnesium sulfate. Copper(I) bromide (CuBr) (Aldrich, St. Louis, MO, USA, 98%) was purified by stirring overnight in glacial acetic acid to remove Cu^2+^, filtered and washed with ethanol, and then dried in vacuo at 70 °C for 2 days. *N*,*N*,*N′*,*N″*,*N″*-Pentamethyldiethylenetriamine (PMDETA) (Aldrich, St. Louis, MO, USA, 99%), α-bromoisobutyryl bromide (Aldrich, St. Louis, MO, USA, 98%), triethylamine (TEA, Aldrich, St. Louis, MO, USA, 99%), dichloromethane (DCM), 1,2-dichloroethane, 2-mercaptoethanol, stannous octanoate Sn(Oct)_2_, and *α*,*α’*-dichloro-*p*-xylene (Acros, Geel, Belgium 98%) were used as received. Conventional procedures were used for the purification of all solvents [68].

### 2.2. Measurement

Transmission IR spectra were recorded on an FT-IR spectrophotometer (Perkin-Elmer 1600, Waltham, MA, USA) in the spectral range 4000–400 cm^−1^ with samples in KBr pellets and NaCl disks. ^1^H and ^13^C NMR spectra were recorded on a Varian Mercury 200 MHz spectrometer (Varian, Palo Alto, CA, USA) and on a Varian Mercury 50 MHz spectrometer (Varian, Palo Alto, CA, USA) with CDCl_3_ as solvent at ambient temperature, respectively. Tetramethylsilane (TMS) was used as the internal standard, and the chemical shifts were given in parts per million (ppm) relative to this standard. GPC analyses were conducted at 30 °C on an Agilent LC GPC (Agilent Technologies, Waldbronn, Germany) system equipped with a refractive index detector, a UV detector, and a set of two columns, ‘Mixed D’ and ‘Mixed E’. THF (HPLC grade) was used as an eluent at a flow rate of 1.0 mL/min for GPC analyses. PMMA standards were used to calibrate the molecular weights. The mass spectra of the synthesized initiator were measured by the electron impact (EI) method with a Micromass Quattro LC-MS/MS spectrophotometer. The thermal properties of the polymers were measured by differential scanning calorimeters (Setaram DSC 141, Laboratory Network, Austin, TX, USA) in a flowing nitrogen atmosphere at 30 °C at a scanning rate of 20 °C/min. The thermal stability of polymers was measured by thermogravimetric analysis (Seiko II Exstar 6000 TG/DTA, Tokyo, Japan) in a flowing nitrogen atmosphere at a heating rate of 20 °C/min and a working temperature range of 30 °C and 600 °C. An electrothermal melting point apparatus (Barnstead-Electrothermal IA9100, Southend-on-Sea, Essex, UK) was used to determine the melting points of the compounds, which are uncorrected. SEM was taken on an Evo MA/LS model of Carl Zeiss electron microscope (Oberkochen, Germany) to investigate the surface morphology of the synthesized macroinitiators and block copolymers. The samples were covered with 100 Å thick gold at 20 mA with the SC7620 Mini Sputter Coater (Quorum, Hertfordshire, UK). Electron images were recorded in a digital environment by transferring the monitor. UV-Vis measurements were carried out with a T80+ UV/VIS spectrometer (PG Instruments, Wibtoft, Leicestershire, UK) using a 1 cm pathlength quartz UV cell.

### 2.3. Synthesis of p-xylene-bis(1-hydroxy-3-thia-propane) (PXTOH)

PXTOH was synthesized by the reaction of *α*,*α′*-dichloro-*p*-xylene with 2-mercaptoethanol in the presence of KOH in ethanol, according to previously published procedures [69,70]. (Yield: 84%, white solid, m.p. 66–68 °C; FT-IR (cm^−1^) (KBr): 3318 (OH), 3042 (ArH), 2949–2814 (CH_2_); ^1^H NMR (CDCl_3_): *δ* = 7.28 (s, 4H, Ar**H**), 3.71 (s, 4H, S–C**H**_2_–Ar), 3.66–3.60 (q, 4H, S–C**H**_2_–CH_2_–OH), 2.66–2.59 (t, 4H, S–C**H**_2_–CH_2_–OH), 2.24 (s, 2H, –O**H**); ^13^C NMR (CDCl_3_): *δ* = 137.2 (**C**_Ar_–CH_2_–S–), 129.1 (**C**H_Ar_), 60.2 (S–CH_2_–**C**H_2_OH), 35.3 (S–**C**H_2_–CH_2_–O), 34.2 (C_Ar_–**C**H_2_–S–). FT–IR (KBr): 3324 (OH), 3057 (ArH), 2954–2827 (CH_2_), 1508, 1475, 1350, 1238, 1089–1045 (OCH_2_), 856, 705, 607 cm^−1^).

### 2.4. Synthesis of 1,4-phenylenebis(methylenethioethane-2,1-diyl)bis(2-bromo-2-methylpropanoate) (PXTBr)

In a 250 mL round bottom two-neck flask equipped with a magnetic stirring bar and an addition funnel, PXTOH (2.58 g, 10 mmol), 4.18 mL (3.04 g, 30 mmol) TEA, and 40 mL anhydrous THF were charged and stirred under a nitrogen atmosphere at room temperature. After cooling to 0 °C, the solution of 3.79 mL α-bromoisobutryl bromide (6.48 g, 30 mmol) in 12 mL THF was added dropwise to the reaction mixture via a dropping funnel at 0 °C over a period of 2 h. The reaction mixture was stirred overnight at room temperature, and the precipitated NHEt_3_Br salt was filtered off. Thin ice particles (25 g) were added to the filtrate, and stirred until the ice melted, and then 50 mL DCM was added to this mixture. After 15 min of stirring at room temperature, the reaction mixture was extracted with 4 × 30 mL of DCM. The organic layer was washed with a 5% NaHCO_3_ solution (2 × 25 mL), dried over anhydrous magnesium sulfate (MgSO_4_) and, filtered, and the DCM removed to obtain a red-brownish viscous product. The yield was 4.67 g (84%).

FT-IR: *υ* (cm^−1^) = 3324 (–OH), 3013 (Ar–H), 2981–2923 (aliphatic–CH_2_), 1734 (C=O), 1272 (–C–O and –C–C), 1159 (asymmetric –C–O–C), 1108 (symmetric –C–O–C). ^1^H NMR (CDCl_3_): δ = 7.28 (s, 4H, Ar**H**), 4.27 (q, 4H, S–CH_2_–C**H**_2_–O–), 3.76 (s, 4H, S–C**H**_2_–Ar), 2.68 (t, 4H, S–C**H**_2_–CH_2_–O–), 1.93 (s, 12H, C(Br)(C**H**_3_)_2_); ^13^C NMR (CDCl_3_): δ = 171.4 (**C**=O), 136.8 (**C**_Ar_–CH_2_–S–), 129.1 (**C**H_Ar_), 64.5 (S–CH_2_–**C**H_2_O), 55.5 (**C**–Br(CH_3_)_2_ 35.9 (C_Ar_–**C**H_2_S–), 30.7 (C–Br(**C**H_3_)_2_), 29.2 (S–**C**H_2_–CH_2_O).

### 2.5. Synthesis of PXTPMMA and PXTPS via ATRP

**Scheme 1:**
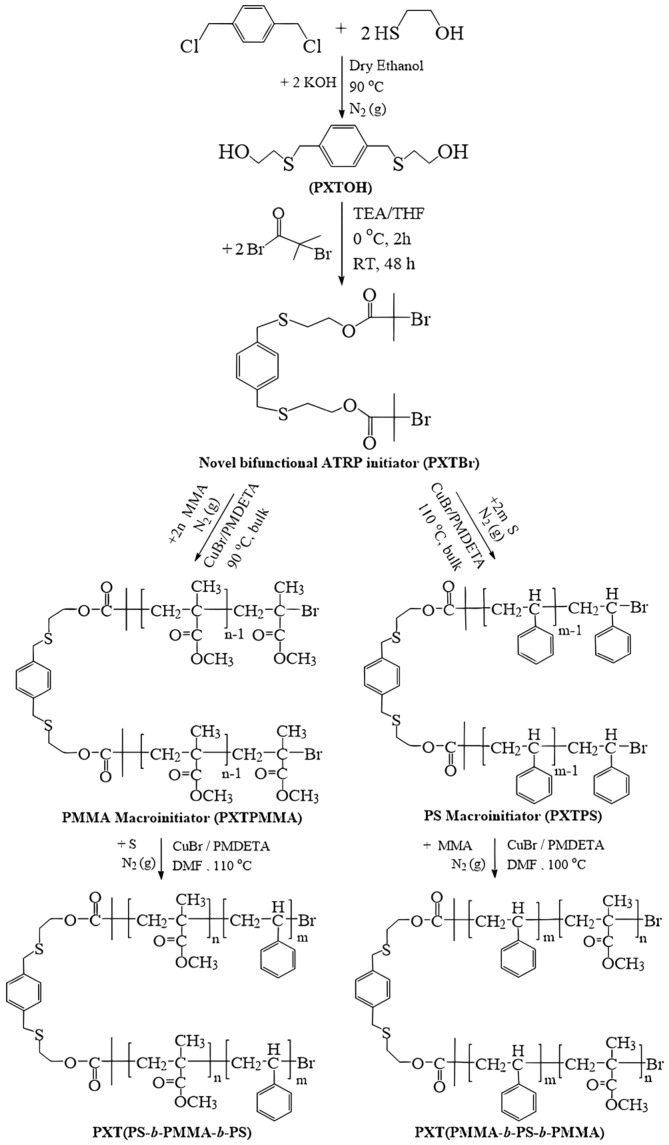
Synthesis of novel ATRP initiator (PXTBr), macroinitiators, and block copolymers.

Scheme 1 shows the reaction pathways of PMMA and PS macroinitiators initiated by PXTBr via ATRP. In a typical bulk polymerization procedure, in a 25 mL Schlenk flask equipped with a magnetic stirrer, the initiator (PXTBr) (0.277 g, 0.5 mmol), MMA (5.38 mL, 50 mmol) or S (5.73 mL, 50 mmol) as monomers, and CuBr (0.146, 1 mmol) as the catalyst were added and stirred under an inert N_2_ atmosphere. After the addition of PMDETA (0.21 mL, 0.2 mmol) as a ligand with deoxygenation by gently purging with nitrogen gas for 20 min to form the Cu complex, the flask was immersed in an oil bath thermostated at 90 °C and 110 °C for MMA and S, respectively. After 0.5 h, the polymerization was quenched by cooling the reaction mixture in an ice bath. The reaction mixture was diluted with THF and passed through a neutral alumina column for the removal of the copper residue. The filtrate was concentrated by rotary evaporation, precipitated into methanol, and filtered off. The obtained polymers were dissolved in CH_2_Cl_2_ and then precipitated two times in cold methanol. The polymers were filtered and dried at 30 °C reduced pressure in a vacuum for 24 h. The conversion, M_n,GPC_, and *Đ* values of PXTPMMA (PM-1) were 21.4%, 6500 g/mol, and 1.28, respectively. For PXTPS (PS-1), these values were 22.8%, 6780 g/mol, and 1.30, respectively.

For kinetics studies, polymerizations were carried out using a mole ratio of [MMA or S]:[CuBr]:[PMDETA]:[PXTBr] = 100:2:2:1 in bulk as described above for additional time intervals such as 1 and 2 h. The conversions of MMA or S were determined gravimetrically. The results and conditions of polymerization for each monomer are given in Table 1.

### 2.6. Synthesis of PXTPCLOH and PXTPCLBr 

**Scheme 2:**
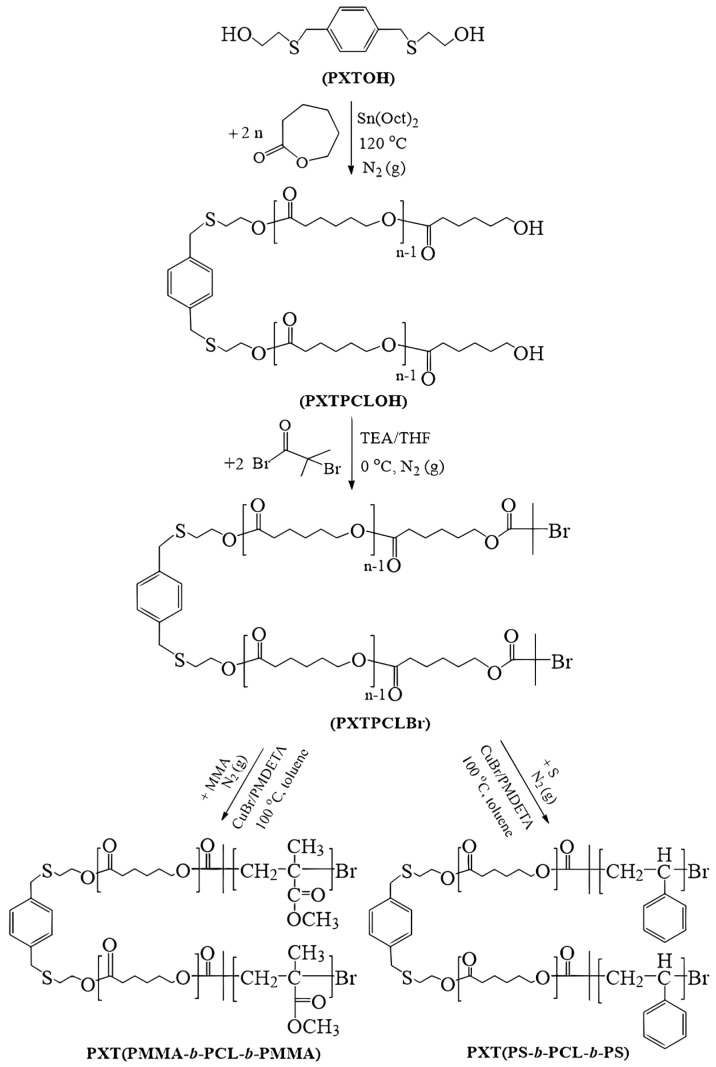
Synthesis of PCL (PXTPCLOH), PCL macroinitiator (PXTPCLBr), and triblock copolymers.

The reaction mechanism is shown in Scheme 2. OH–PCL–OH (PXTPCLOH) was synthesized via ROP of *ε*-CL as follows: the ROP initiator (PXTOH) (0.52 g, 2 mmol), *ε*-CL (9.13 g, 80 mmol), and Sn(Oct)_2_ (3.41 × 10^−3^ mL, 0.01 mmol) were introduced into the 25 mL Schlenk flask equipped with a magnetic stirrer. The reaction mixture was stirred and purged with nitrogen at room temperature. The flask was then placed into a preheated constant–temperature oil bath at 120 °C for 24 h. The crude polymer was dissolved in a small amount of DCM and then precipitated into an excess of cold methanol. The resulting polymer was dried in the vacuum oven at room temperature until it reached a constant weight. M_n,GPC_ and Đ values of the polymer are 8620 g/mol and 1.29, respectively.

PXTPCLBr was synthesized by using α-bromoisobutyrl bromide in the presence of TEA in THF. A 100 mL round bottom flask was charged with PXTPCLOH (3.5 g, 0.41 mmol, M_n_ = 8620 g/mol), which was dissolved in dry THF (30 mL) with TEA (0.16 g, 1.64 mmol) while stirring under a nitrogen atmosphere. α-bromoisobutyrl bromide (0.21 mL 0.37 g, 1.64 mmol) in dry THF (15 mL) was added dropwise to the cooled reaction mixture at 0 °C for 30 min. The reaction mixture was stirred at room temperature for 48 h. The precipitated salt was removed by filtration, and then the filtrate was evaporated. Thin ice particles were added to the crude product and then to 120 mL of DCM. The organic phase was washed successively with (4 × 30 mL) DCM and (2 × 25 mL) 5% aqueous NaHCO_3_ and finally dried over MgSO_4_. The concentrated solution was precipitated into excess cold methanol, and the product was dried in a vacuum. M_n,GPC_ is 8950 g/mol, *Đ* is 1.33.

### 2.7. The Synthesis of Block Copolymers

The synthesis of block copolymers was accomplished by ATRP of S and MMA in DMF with CuBr/PMDETA as a catalyst using the previously obtained PXTPMMA (PM-1) and PXTPS (PS-1) as the macroinitiators, respectively. The polymerizations were carried out at 110 °C (for S) and at 100 °C (for MMA) with the mol ratio of [S or MMA]:[CuBr]:[PMDETA]:[PXTPMMA or PXTPS] = 1000:2:2:1. Block copolymerization was performed according to the same procedure used for the synthesis of macroinitiators. For the synthesis of PXT(PS-*b*-PMMA-*b*-PS) triblock copolymer (ABA–1), S (5.73 mL, 50 mmol), PXTPMMA (0.65 g, 0.1 mmol), DMF as solvent (volume ratio of monomer–solvent = 1:3), and PMDETA (42 µL, 0.2 mmol) were added to the Schlenk tube sealed with a rubber septum and vacuum-N_2_. After four freeze–pump cycles to remove oxygen, CuBr (28.6 mg, 0.2 mmol) was added to the tube and mixed for 10 min to form the complex. The flask was immersed in an oil bath thermostated at 110 °C for 24 h. After this time, the polymerization was quenched by cooling the reaction mixture in an ice bath, and the mixture was diluted with THF and passed through a neutral alumina column for the removal of the copper residue, which precipitated into methanol. The obtained light-yellow product was dissolved in THF, precipitated in cold methanol. The PXT(PS-*b*-PMMA-*b*-PS) was collected after filtration and dried at room temperature for 48 h in a vacuum. The conversion, M_n_,_GPC_ and *Đ* values of S with ATRP reaction were found to be 26.6%, 4.6 × 10^4^ g/mol, and 1.52, respectively. The conversion, M_n,GPC_, and *Đ* values of MMA by ATRP reaction were 23.2%, 3.12 × 10^4^ g/mol, and 1.34, respectively.

PXT(PMMA-*b*-PS-*b*-PMMA) (ABA–2), was achieved by ATRP of MMA (5.36 mL, 50 mmol) using PMDETA (42 µL, 0.2 mmol), CuBr (28.6 mg, 0.2 mmol), DMF (MMA/DMF = 3 (*v*/*v*)), and PXTPS (0.68 g, 0.0156 mmol) under degassed conditions at 100 °C for 24 h. The polymer was obtained as described above for the PXT(PS-*b*-PMMA-*b*-PS) copolymer. The polymerizations were also carried out at the same temperature and catalyst system with a mole ratio of [S or MMA]:[CuBr]:[PMDETA]:[PXTPMMA or PXTPS] = 500:2:2:1, and all polymerization conditions and results are given in detail in Table 2.

PCL-based block copolymers were synthesized by ATRP of S and MMA in toluene with CuBr/PMDETA as catalyst systems using the previously obtained PXTPCLBr, respectively. The polymerizations were carried out at 100 °C for both S and MMA according to the same procedure used for the synthesis of block copolymers as described above with the mol ratio of [S or MMA]:[CuBr]:[PMDETA]:[PXTPCLBr] = 1000:2:2:1. For the synthesis of PXT(PS-*b*-PCL-*b*-PS) and PXT(PMMA-*b*-PCL-*b*-PMMA) triblock copolymer (ABA–5 and ABA–6), S or MMA (50 mmol), PXTPCLBr (0.447 g, 0.05 mmol)), toluene as solvent, and PMDETA (21 µL, 0.1 mmol) were added to the Schlenk tube sealed with a rubber septum and vacuum–N_2_. After four freeze-pump-cycles to remove oxygen, CuBr (14.3 mg, 0.1 mmol) was added to the tube and mixed for 10 min to form the complex. The flask was immersed in an oil bath thermostated at 100 °C for 24 h. After this time, the polymerization was quenched by cooling the reaction mixture in an ice bath, and the mixture was diluted with THF and passed through a neutral alumina column for the removal of the copper residue, which precipitated into methanol. The obtained product was dissolved in CH_2_Cl_2_, and precipitated in cold methanol. The block copolymer was collected after filtration and dried at room temperature for 48 h in a vacuum. For PXT(PS-*b*-PCL-*b*-PS), the conversion is 60.5%, M_n,GPC_ is 13.15 × 10^4^ g/mol, and *Đ* is 1.21. For PXT(PMMA-*b*-PCL-*b*-PMMA), the conversion is 67.5%, M_n,GPC_ is 8.28 × 10^4^ g/mol, and *Đ* is 1.40.

### 2.8. Measurement of the Heavy–Metal–Binding Properties of the PCL and PS

The extraction properties of PXTPCLOH and PXTPS were investigated under liquid–liquid phase conditions using heavy metal picrates (Ag^+^, Cd^2+^, Cu^2+^, Hg^2+^, and Pb^2+^) as substrates by measuring with UV-Vis the amounts of picrate in the 1,2-dichloroethane phase before and after treatment with polymers. The extraction of metal picrates from the aqueous phase to the 1,2-dichloroethane phase by the synthesized PCL and PS was examined by liquid–liquid extraction.

Polymer solutions of 3.0 × 10^−4^ M were prepared by dissolving 194 mg of PCL (PXTPCLOH) or 196 mg of PXTPS (Entry PS–2, in Table 1), whose molar masses (GPC) are close to each other, in 1,2-dichloro ethane. The heavy metal picrate solutions were prepared by placing 5 mL of metal nitrate solution (3.5 × 10^−4^ M) and 5 mL of picric acid (1.5 × 10^−3^ M) solution into test tubes for each metal and keeping them in the dark for a few hours. (Heavy metal picrate solutions were prepared by the mixture of metal solution (3.5 × 10^−4^ M) and 5 mL of picric acid (1.5 × 10^−3^ M) solution in water.) The UV–Vis spectra of polymers (PXTPCLOH and PXTPS) and metal picrate solutions were obtained using the measurement of absorbance values. The metal picrate solutions (10 mL) and polymer solutions (10 mL) for each one were put in 12 glass bottles and shaken vigorously for 48 h at room temperature for each metal picrate and polymer. After shaking, the 1,2-dichloroethane phase was separated, and the percent of picrate salt extracted (*E*%) was determined by UV–Vis spectrophotometry.

(1) $$E\left(\%\right)=\left(\frac{A_{before}-A_{after}}{A_{before}}\right)\times 100$$

*A_before_* is the absorbance of absence of polymers. *A_after_* denotes the absorbance in the 1,2-dichloroethane phase mixture.

## 3. Results and Discussion

Triblock copolymers were synthesized by chain extension of PS, PMMA, or PCL macroinitiators initiated by the novel ATRP (PXTBr) and ROP (PXTOH) initiators. Firstly, PXT(PS-*b*-PMMA-*b*-PS) and PXT(PMMA-*b*-PS-*b*-PMMA) ABA–type triblock copolymers were synthesized in four steps: the synthesis of diol compounds (PXTOH) (i), a novel bifunctional ATRP initiator (PXTBr) (ii), PMMA and PS macroinitiators (PXTPMMA and (PXTPS) (iii), and ABA–type triblock copolymers (iv). In the first step, (PXTOH) was synthesized according to the literature [69] by the reaction of *α*,*α’*-dichloro-*p*-xylylene with 2-mercaptoethanol in the presence of KOH/ethanol media. In the second step, a novel bi-functional initiator (PXTBr) was prepared by the esterification of α-bromoisobutryl bromide with PXTOH for ATRP initiation. In the third step, PMMAs and PSs as macroinitiators were prepared via ATRP with a CuBr/PMDETA catalyst system initiated by compound PXTBr. In the final step, these macroinitiators were used to obtain ABA-type block copolymers by the ATRP method.

We also synthesized PCL-based PS and PMMA block copolymers using the same diol initiating compound with a combination of ROP and ATRP. For this goal, ABA-type triblock copolymers consisting of (PS) or (PMMA) as side segments and (PCL) as middle segments were also synthesized via the combination of (ROP) and (ATRP). For this purpose, telechelic PCL-diol(PXTPCLOH) was synthesized by ROP of *ε*-CL in bulk at 120 °C using PXTOH as the difunctional initiator and Sn(Oct)_2_ as the catalyst. Di(α-bromoester) end–functionalized PCL (PXTPCLBr) was prepared by esterification of hydroxyl end groups of PXTPCLOH with α-bromoisobutyryl bromide in the presence of TEA in THF. ABA–type block copolymers, PXT(PMMA-*b*-PCL-*b*-PMMA) and PXT(PS-*b*-PCL-*b*-PS), were synthesized by ATRP of MMA and S as monomers using PXTPCLBr and CuBr/PMDETA as catalyst systems in toluene, respectively.

### 3.1. The Characterization of PXTBr (ATRP Initiator)

**Figure 1:**
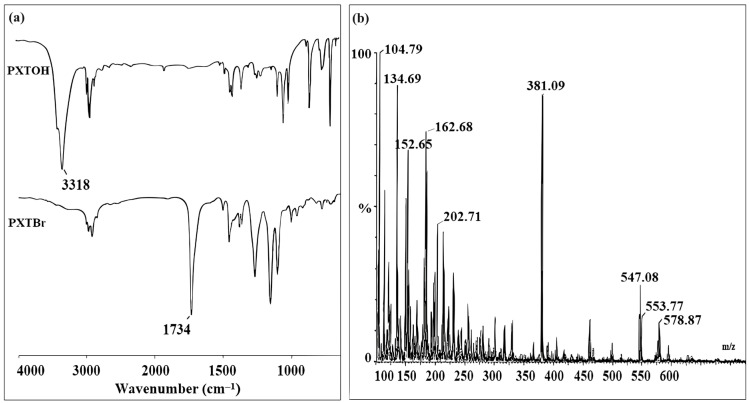
(**a**) FT-IR spectra of (PXTOH) and (PXTBr), (**b**) ESI mass spectrum of (PXTBr).

The synthetic approach for the preparation of the novel bifunctional initiator (PXTBr) is illustrated in Scheme 1. The novel ATRP initiator (PXTBr) was characterized by FT-IR, ESI-MS, ^1^H NMR, and ^13^C NMR. FT-IR spectra of PXTOH and PXTBr are shown in Figure 1a. The O–H stretching vibration as a strong broad absorption at 3318 cm^−1^ in the FT-IR spectrum of compound PXTOH disappeared after the esterification, and the appearance of the characteristic absorption peak at 1734 cm^−1^, which was ascribed to the C=O (ester carbonyl group) in the IR spectrum, confirmed the formation of the novel initiator PXTBr. The peaks at 2981 and 2923 cm^−1^, assigned to the C–H symmetric and asymmetric vibration (the –CH_2_– and –CH_3_ groups), respectively, and the peaks at 1272 and 1159 cm^−1^ due to the C–O stretching band in the spectrum, were also observed in Figure 1a as a result of esterification. The mass spectral data of PXTBr (Figure 1b) showed a peak of *m/z* = 553.77 corresponding to [M]^+^. The spectrum also showed a peak of *m/z* = 578.87 corresponding to [M+Na+2H]^+^, which was in accordance with the theoretical calculation.

**Figure 2:**
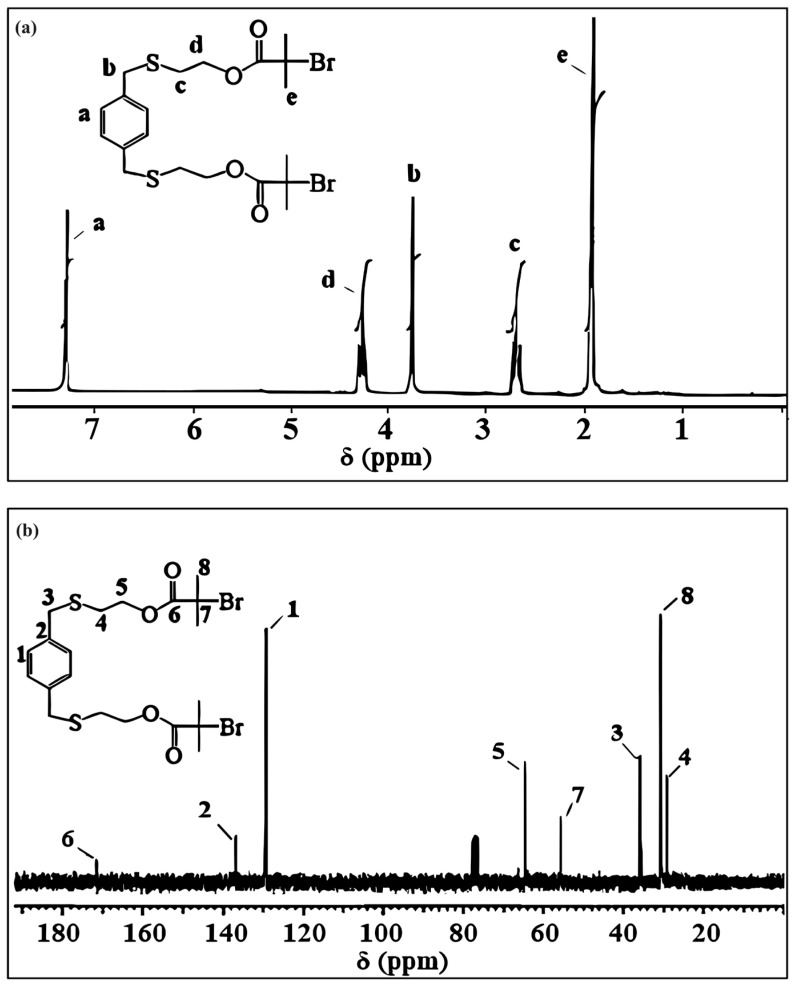
(**a**) ^1^H NMR and (**b**) ^13^C NMR spectrum of PXTBr.

The ^1^H NMR and ^13^C NMR spectra of PXTBr, along with assignments, are shown in Figure 2. In the ^1^H NMR spectrum (Figure 2a), the peak at δ = 2.24 ppm, corresponding to the primary –O–H groups’ protons, disappeared in comparison to the ^1^H NMR spectrum(data) of PXTOH. The protons (H^e^) of methyl groups (C(Br)(CH_3_)_2_) appeared as a singlet at δ = 1.93 ppm as a result of esterification in PXTBr (Figure 2a). Hill et al. showed that the protons (H^e^) of the methyl groups (C(Br)(CH_3_)_2_) occured singlet at δ = 1.93 ppm as a result of esterification of the difunctional initiator [71]. The triplets appearing at δ = 4.27 and 2.69 ppm in the spectrum (Figure 2a) were assigned to methylene protons (SCH_2_CH_2_O) and (SCH_2_CH_2_O), respectively. The spectrum also gave signals at δ = 3.67 ppm for benzylic (ArCH_2_S) and at δ = 7.28 ppm for the aromatic protons of PXTBr. As seen in Figure 2b, the ^13^C NMR spectrum of PXTBr clearly indicated the presence of carbonyl, quaternary, and methyl carbon atoms of the bromoisobutyryl end group at δ = 171.4 (C6) (C=O), 55.5 (C7) (C–Br(CH_3_)_2_, and 30.7 (C8) (C–Br(CH_3_)_2_), respectively. The aromatic carbon signals of PXTBr were observed at 129.2 (C1) and 136.8 (C2) ppm. ^13^C NMR spectra also indicated the methylene carbon signals of the PXTBr were at 64.5 ppm (SCH_2_CH_2_O), 35.9 ppm (ArCH_2_S), and 29.2 ppm (SCH_2_CH_2_O) (Figure 2b). All spectral data were in good agreement with the novel synthesized ATRP initiator (PXTBr).

### 3.2. The Characterization of Macroinitiators (PXTPMMA and PXTPS) and Their Block Copolymers

The macroinitiators, PXTPMMAs (PM1–3, in Table 1) and PXTPSs (PS1–3, in Table 1), were synthesized by ATRP in bulk using a novel bifunctional initiator (PXTBr) in the presence of CuBr/PMDETA as a catalyst system at 90 °C and 110 °C, respectively (Scheme 1). The obtained PMMA and PS macroinitiators were used to obtain ABA-type block copolymers by the ATRP method in DMF. The polymerization results and conditions for different reaction times are summarized in Table 1. The conversions were calculated gravimetrically. The macroinitiators and block copolymers were characterized by FT-IR, ^1^H NMR, GPC, DSC, TGA, and SEM.

**Figure 3:**
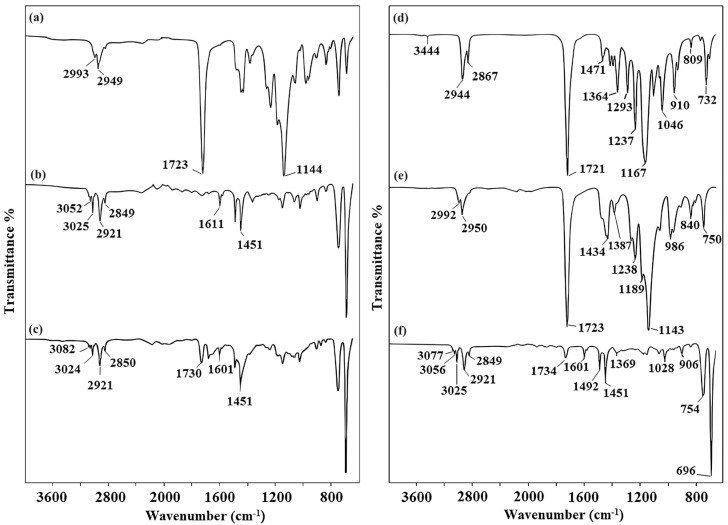
FT-IR spectra of the macroinitiators and block copolymers. (**a**) PXTPMMA, (**b**) PXTPS, (**c**) PXT(PS-*b*-PMMA-*b*-PS), (**d**) PXTPCLOH, (**e**) PXT(PMMA-*b*-PCL-*b*-PMMA), (**f**) PXT(PS-*b*-PCL-*b*-PS).

FT-IR spectra of the macroinitiators (PXTPMMA and PXTPS) and their block copolymers are shown in Figure 3. As seen in Figure 3a, the characteristic absorption peak of the carbonyl group at around 1723 cm^−1^ with high relative intensity is PMMA block units, indicating the polymerization of MMA initiated by the PXTBr. The aromatic C–H stretchings and aliphatic C–H stretches were monitored at 3052–3025 cm^−1^ and 2921–2849 cm^−1^ for the PS macroinitiator (PS-1) (Figure 3b). In Figure 3c, the appearance of the peaks at 3082–3025 and 1601 cm^−1^ of PS units and the peaks at 1730 cm^−1^ corresponding to PMMA units indicated that a block copolymer (ABA–1) was formed.

**Figure 4:**
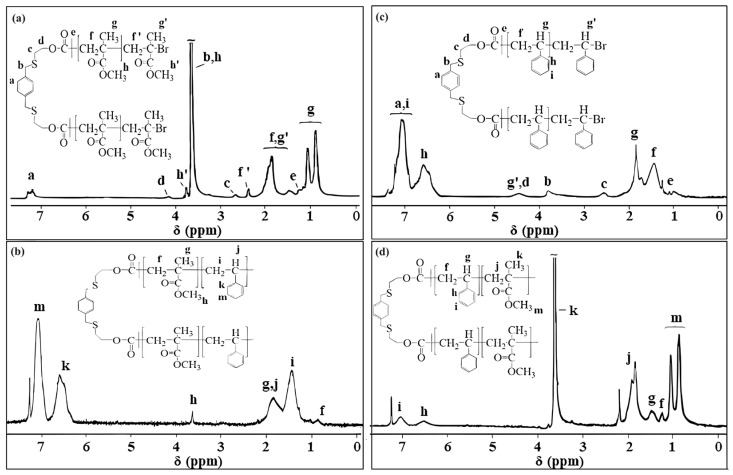
^1^H NMR spectra of macroinitiators and their block copolymers (PM-1), (PS-1), (ABA–1), and (ABA–2) are indicated in (**a**), (**c**), (**b**), and (**d**), respectively.

The ^1^H NMR spectra (Figure 4a,b) of PXTPMMA and PXT(PS-*b*-PMMA-*b*-PS) are shown with the characteristic protons of the main segments. The ^1^H NMR spectrum (Figure 4a) of PXTPMMA showed the attributes of the peaks of the polymer and the initiator fragment. The resonance peaks of methyloxy groups at 3.67 ppm (s, –OC**H**_3,_ H^h^), methylene groups at 1.80–2.07 (m, –C**H**_2_, H^f^), and methyl groups at 0.84–1.10 (m, –C**H**_2_, H^g^) of the PMMA main chain were detected with high resolution. The signals assigned to methyl protons ((C(Br)(C**H**_3_)_2_), H^e^) shifted from 1.93 ppm (in Figure 2a) to 1.17 ppm as a result of polymerization. On the other hand, the signals in the ^1^H NMR spectrum belonging to methylene protons of initiator fragment at 4.18 (SCH_2_C**H**_2_O_,_ H^d^) and 2.69 ppm (SC**H**_2_CH_2_O, H^c^) proved the polymerization of MMA initiated by PXTBr. ^1^H NMR spectra also indicated methyloxy (H^h′^), the methylene (H^f’^) and methyl (H^g’^) group protons of the terminal PMMA segment. The peak originated from benzylic (ArC**H**_2_S) protons of the initiator and could not be observed since the peak of methoxy groups at 3.67 ppm corresponding to the polymer backbone overlapped this peak.

The ^1^H NMR spectrum (Figure 4b) of PXT(PS-*b*-PMMA-*b*-PS) displayed characteristic signals in the range of 7.27–6.41 ppm and 1.21–2.18 ppm, respectively, assigned to the aromatic and aliphatic proton of the PS main chain. The signals at 3.67 ppm and 0.92 ppm were ascribed to the methoxy and methylene protons of the PMMA segment, respectively.

The ^1^H NMR spectra (Figure 4c,d) of PXTPS and PXT(PMMA-*b*-PS-*b*-PMMA) are shown with the characteristic protons of the main segments. ^1^H NMR (in CDCl_3_) spectra (Figure 4c) of PXTPS showed characteristic peaks in the region of 6.31 to 7.32 ppm, which correspond to aromatic protons (H^h+i^) of PS and aromatic protons (H^a^) of PXTBr. The peaks in the region of 1.11 to 2.52 ppm are attributable to the protons of methine (H^g^) and methylene (H^f^) groups in the main chain of PS. The overlapping signals in the range 4.22–4.64 ppm appeared due to methine protons (H^g’^) to which bromine is attached, and methylene protons (H^d^), attached to the ester group, respectively. On the other hand, the signals of methylene protons in the initiator fragment were observed at 3.75 ppm (ArCH_2_SC**H**_2,_ H^b^) and 2.69 ppm (SC**H**_2_CH_2_O, H^c^). ^1^H NMR spectrum (Figure 4d) exhibited new peaks at 3.67 ppm and 0.92 ppm corresponding to methoxy and methylene protons, respectively, of the PMMA segment in addition to the other characteristic peaks of PS at 6.3–7.3 and 1.1–1.7 ppm, confirming the formation of the block copolymer PXT(PMMA-*b*-PS-*b*-PMMA)).

As shown in Table 1 the number-average molecular weight (M_n_) of the macroinitiators increased with conversion and time during the polymerizations of both MMA and S. Polymerizations of MMA in 0.5 h and 1 h resulted in conversion (21.4 and 55.3%) and narrow polydispersities (*Đ*) (1.28 and 1.31), respectively. Table 1 also shows that the conversion in 0.5 h and 1 h remained low (22.4 and 25.7%) and with a narrow *Đ*s (1.30–1.34) for the ATRP of S. However, *Đ* broadened to 1.75 and 1.49 at higher conversions of MMA and S, respectively, compared to previous entries, indicating poor control in the 2 h polymerization of MMA and S. The initiator efficiency (*f*) of macroinitiators varied between 0.34 and 0.43. The theoretical and experimental number-average molecular weights M_n,th_ and M_n,GPC_ are not close enough to each other, and the initiator efficiencies (*f*) of macroinitiators decrease during the polymerization. These can be attributed to defects such as slow initiation and termination reactions [72] Guan et al. discovered that when a small quantity of S is added to PMMA–Cl, S performs its initiation rapidly, the reaction ends quickly, and the block copolymer’s molecular weight has a wide distribution [73].

**Figure 5:**
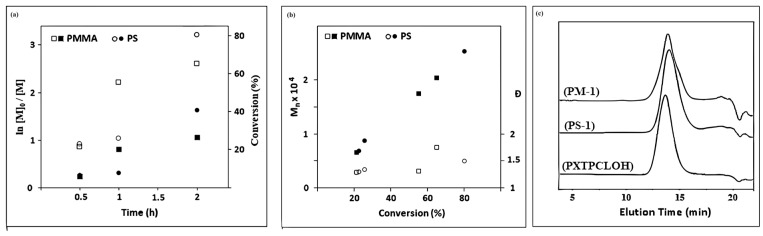
(**a**) Kinetic plot of conversion and ln[M]_0_/[M]_t_ vs. time and (**b**) plot of (M_n,GPC_) and *Ð* vs. conversion (%) for ATRP of MMA and S. (**c**) GPC traces of macroinitiators.

Kinetics studies of ATRP of MMA and S using PXTBr as the initiator with [MMA or S]:[I]:[CuBr]:[PMDETA] = 100:1:2:2 in bulk at 90 °C were carried out. The constant concentration of active species during polymerization was proved by the linear relationship between ln [M]_0_/[M] and time (Figure 5a). The linearity of the plot of molecular weight (M_n,GPC_) vs. conversion percentage and polydispersity index for both macroinitiators also demonstrated controlled polymerization behavior. The semilogarithmic kinetic plots of ln([M]_0_/[M]) versus time for ATRP of MMA and S using PXTBr as initiator in bulk are presented in Figure 5b. GPC curves of PMMA and PS macroinitiators (PM-1 and PS-1) (in 0.5 h) showed low *Đ*s** (<1.35) and narrow unimodal M_n_ distributions (Figure 5c).

**Figure 6:**
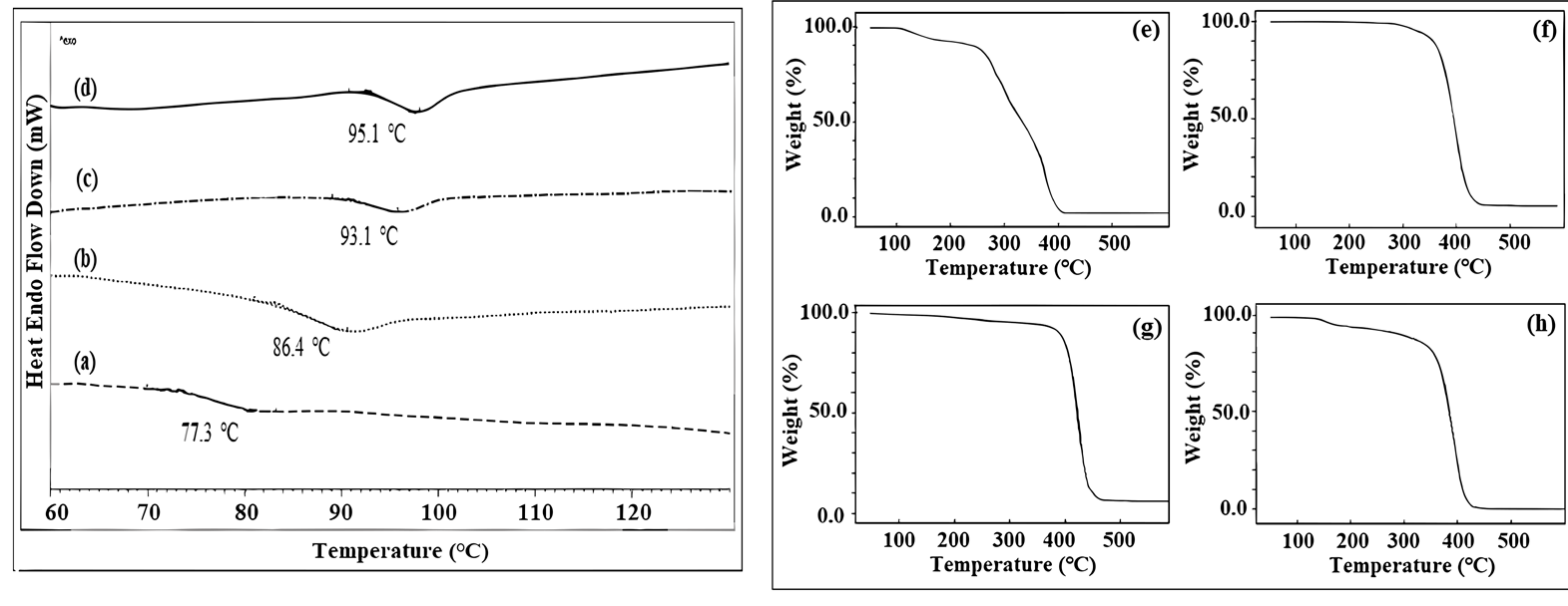
DSC traces and TGA curves of PXTPMMA and PXTPS macroinitiators and their block copolymers. (**a**) PXTPMMA, (**b**) PXTPS, (**c**) PXT(PMMA-*b*-PS-*b*-PMMA), (**d**) PXT(PS-*b*-PMMA-*b*-PS), (**e**) PXTPMMA, (**f**) PXTPS, (**g**) PXT(PS-*b*-PMMA-*b*-PS), (**h**) PXT(PMMA-*b*-PS-*b*-PMMA).

The thermal properties of the synthesized polymers were analyzed by differential scanning calorimetry (DSC) and thermal gravimetric analysis (TGA). The macroinitiators and block copolymers presented a phase transition behavior corresponding to that of amorphous PS and PMMA polymers. The glass transition temperatures (*T*_g_s) of PXTPS, PXTPMMA, and their block copolymers were examined by DSC under a nitrogen atmosphere. The *T*_g_ values of PXTPMMA (Table 1, entry PM-1) and PXTPS (Table 1, entry PS-1) were found to be 77.3 and 86.4 °C, respectively (Figure 6a,b). For PXT(PMMA-*b*-PS-*b*-PMMA) (Table 2, entry ABA–2) in Figure 6c, one glass transition region is observed, *T*_g_, at 93.17 °C, associated with PMMA–rich regions. In Figure 6d, there is also only one *T*_g_ at 95.11 °C for PXT(PS-*b*-PMMA-*b*-PS) (Table 2, entry ABA–1). This behavior could be attributed to the length of each block in the ABA-type copolymers.

The TGA curves obtained for the macroinitiators, PXTPMMA and PXTPS, and their block copolymers, are shown in Figure 6e–h. As shown in Figure 6e, TGA analysis of PXTPMMA (Table 1, PM-1) shows a three–step thermal decomposition. The TGA curve exhibited three weight losses at approximately 175 °C, 307 °C, and 395 °C. The second weight change is attributable to the decomposition of material at 265–330 °C. In Figure 6f, PXTPS (Table 1, PS-1) has a decomposed maximum point at 402 °C for PS. PXT(PS-*b*-PMMA-*b*-PS) (Table 1, ABA–1) shows one weight loss at 426 °C in Figure 6g. In Figure 6h, the TGA curve for PXT(PMMA-*b*-PS-*b*-PMMA) exhibits two maximus points at 172 and 395 °C.

**Figure 7:**
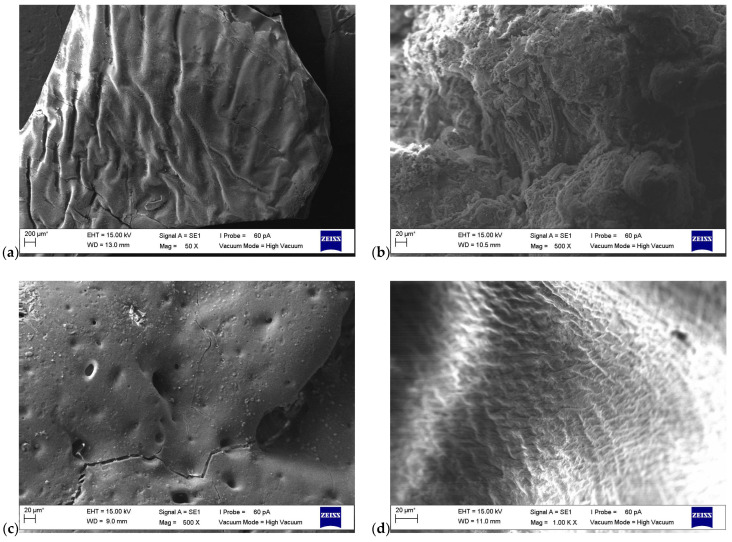
SEM images of (**a**) PXTPMMA (PM-1) (**b**) PXTPS (PS-1), (**c**) PXT(PMMA-*b*-PS-*b*-PMMA) (ABA–2), (**d**) PXT(PS-*b*-PMMA-*b*-PS) (ABA–1).

SEM images of the macroinitiators and the block copolymers are given in Figure 7a–d. From the SEM image of PXTPMMA (Figure 7a), the two-armed branched PMMA blocks are a homogeneous arrangement on the surface. At the same time, there are folds and crevices on the surface. The surface shows a smooth and regular chain arrangement in some regions. SEM microfilm of PXTPS (Figure 7b) shows that the PS blocks in the macroinitiator branch on the surface are relatively short, two-armed blocks. There are very small micropores in this polymeric structure. On the surface, the branching resembles the distribution of spherical micelles, but this distribution is not homogeneous and regular. In Figure 7c, it can be seen that the surface film of PXT(PMMA-*b*-PS-*b*-PMMA) is rough and has macro- and micropores. SEM microfilm of the PXT(PS-*b*-PMMA-*b*-PS) block copolymer is given in Figure 7d. In the SEM image of PXT(PS-*b*-PMMA-*b*-PS), the block copolymer surfaces show sharp, curved, fibrous shapes. These filamentous chains are arranged horizontally and vertically on the surface.

### 3.3. The Characterization of PXTPCLOH and PXTPCLBr

PCL-based triblock copolymers were obtained in three stages: ROP of *ε*-CL (i), esterification of PCL-diol (ii), and polymerization of MMA and S via ATRP (iii). Dihydroxyl-terminated PCL ([monomer]/[initiator] = 40) was synthesized by ROP of *ε*-CL, as the monomer, initiated by PXTOH in the presence of Sn(Oct)_2_ at 120 °C for 24 h. PXTPCLOH was characterized by FT-IR, ^1^H NMR, GPC, DSC, and TGA. FT-IR spectra of PXTPCLOH, PXTPCLBr, and block copolymers are shown in Figure 3d–f. As seen in Figure 3d, the characteristic absorption peak of the carbonyl group at around 1721 cm^−1^ with high relative intensity, arising from the formation of the ester bond, is PCL block units, indicating the polymerization of *ε*-CL. The bands at 1471 and 1364 cm^−1^ represent the stretching of C–H in the methylene groups of PCL. Other characteristic bands of the PCL block are observed at 1293, 1237, 1167, 1046, and 960 cm^−1^. These bands belong to stretching vibrations (–C–O and –C–C), asymmetric –C–O–C) (symmetric –C–O–C), and C–C stretching, respectively [53,74]. The spectrum also showed two bands at 2944–2867 cm^−1^, representing symmetric and asymmetric CH_2_ stretching, respectively. The band observed at 732 cm^−1^ was assigned to the CH_2_ rocking vibration of PCL, which indicates a linear chain aliphatic PCL structure [75,76,77].

**Figure 8:**
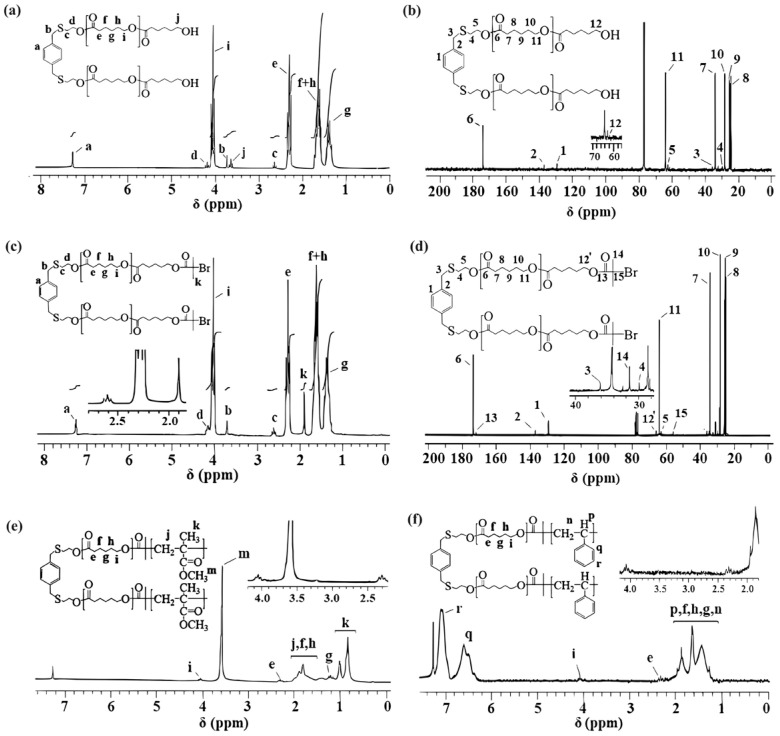
^1^H NMR spectra of (**a**) PXTPCLOH (**c**) PXTPCLBr and ^13^C NMR spectra of (**b**) PXTPCLOH (**d**) PXTPCLBr PXTPCLOH, and ^1^H NMR spectra of (**e**) PXT(PMMA-*b*-PCL-*b*-PMMA) (**f**) PXT(PS-*b*-PCL-*b*-PS).

The ^1^HNMR and ^13^CNMR spectra of PXTPCLOH along with assignments, are shown in Figure 8a,b. In the ^1^H NMR spectrum (Figure 8a) of PXTPCLOH in CDCl_3_, the peak at δ = 2.24 ppm, corresponding to the primary –O–H groups’ protons, disappeared in comparison to the ^1^H–NMR spectrum of PXTOH. δ = 4.18 (t, S–CH_2_–C**H**_2_–O, H^d^), 4.06 (t, CH_2_C**H**_2_–O–, H^i^), 3.76 (s, Ar–C**H**_2_–S–CH_2_–CH_2_–O, H^b^), 2.65 (Ar–CH_2_–S–C**H**_2_–CH_2_–O, H^c^), 2.30 (t, O=C–C**H**_2_CH_2_–, H^e^), 1.63 (m, –OCH_2_C**H**_2_CH_2_C**H**_2_CH_2_COO–, H^f+h^), and 1.36 (m, OCH_2_CH_2_C**H**_2_CH_2_CH_2_COO–, H^g^). The observation of the signal of the primary hydroxymethylene end group (t –CH_2_C**H**_2_–OH) of repeating PCL at 3.65 ppm indicates that the block has living character. The signals assigned to methylene protons δ = 4.18 (t, S–CH_2_–C**H**_2_–O, H^d^) are observed as a triplet at 2.33 (t, S–CH_2_–C**H**_2_–O, H^c^) ppm, as a result of polymerization in PCL. The peaks at 7.28 (Ar–**H**, H^a^) and 3.71 (Ar–C**H**_2_S, H^b^) ppm are attributed to the aromatic and benzylic methylene protons in the initiator PXTOH fragment, respectively.

As seen in Figure 8b, in the ^13^C NMR spectrum of PXTPCLOH, the carbon resonance of the characteristic carbonyl of the repeating unit of PCL is observed at 173.7 (C6) ppm, and the chemical shift values of aliphatic protons are at 64.2 (C11), 34.1 (C7), 25.2 (C10), 25.1 (C9), and 24.7 (C8) ppm, respectively. The signals of aromatic carbons for initiator PXTOH were observed at 137.7 (C1) and 129.4 (C2) ppm, respectively. The peaks at 63.7 (S–CH_2_–**C**H_2_O, C5), 35.9 (Ar–**C**H_2_–S, C3), and 29.1 (S–**C**H_2_–CH_2_O–, C4) ppm were attributed to the methylene groups of initiator PXTOH. The signal at 62.4 (**C**H_2_OH, C12) ppm methylene protons adjacent to the hydroxyl end group of PCL is also seen in Figure 8b. [78] These observations indicated that PXTPCLOH was successfully synthesized and that *ε*–CL monomers were inserted into the SCH_2_CH_2_OH bonds of the bifunctional initiator (PXTOH) through the selective acyl-oxygen cleavage of the monomer [53].

The second step was the synthesis of PXTPCLBr by chemical modification of PXTPCLOH. OH–PCL–OH was esterified by α-bromoisobutyryl bromide to allow the transformation of the hydroxyl-ended groups of PCL into the bromine-containing ATRP initiation groups. The PCL macroinitiator was characterized by ^1^H NMR (Figure 8c) and ^13^C NMR (Figure 8d). After esterification of the hydroxyl groups of PCL, methylene protons adjacent to hydroxyl end groups (H^j^ in Figure 8a) shifted from 3.65 to 4.08 ppm [79]. A new sharp single peak (H^k^) appeared at 1.90 ppm, corresponding to the methyl groups adjacent to the bromine atom. No signal was found at or near 3.65 ppm after esterification, demonstrating that PCL had been completely converted to a bromo-ester derivate [59]. The resonance peaks of repeating methylene protons of the PCL main chain at 4.06 (t, CH_2_CH_2_–O–, H^i^), 2.30 (t, O=C–CH_2_CH_2_–, H^e^), 1.63 (m, –OCH_2_CH_2_CH_2_CH_2_CH_2_COO–, H^f+h^), and 1.36 (m, OCH_2_CH_2_CH_2_CH_2_CH_2_COO–, H^g^) ppm were detected with high resolution. The signals of PCL–bromoester assigned to methylene protons(–S–CH_2_–CH_2_–O), (Ar –CH_2_–S), and (–S–CH_2_CH_2_–O–) shifted from 4.18 to 4.16 ppm, 3.76 to 3.70, and 2.65 ppm to 2.60 ppm, respectively, as a result of esterification.

As seen in Figure 8d, in the ^13^C NMR spectrum of PXTPCLBr, the carbon resonance of the characteristic carbonyl of the repeating unit of PCL is observed at 173.7 (C6) ppm, and the chemical shift values of aliphatic protons are at 64.3 (C11), 34.1 (C7), 25.7 (C10), 25.5 (C9), and 24.7 (C8) ppm, respectively. According to Figure 8d, the ^13^C NMR spectrum of PXTPCLBr clearly indicates the presence of carbonyl, quaternary, and methyl carbon atoms of the bromoisobutyryl end group at 171.8 (C13) (**C**=O), 56.1 (C15) (**C**–Br(CH_3_)_2_, and 33.5 (C14) (C–Br(**C**H_3_)_2_) ppm, respectively. The signals of aromatic carbons for initiator PXTOH are observed at 137.6 (C2) and 129.1 (C1) ppm. The benzylic methylene carbon initiator is 35.9 (C_Ar_–**C**H_2_S–, C4) ppm. The peaks of methylene groups of PXTOH are at 63.4 (–SCH_2_**C**H_2_O, C5), 36.1 (Ar**C**H_2_S**C**H_2_O, C3), and 29.2 (SCH_2_CH_2_, C4) ppm. The signal of methylene protons adjacent to the oxygen–end group of the PCL main chain shifted from 62.4 to 64.8 (**C**H_2_OH, C12′) ppm as a result of esterification.

### 3.4. The Characterization of Block Copolymers Using PXTPCLBr via ATRP

The synthesis of block copolymers was carried out at 100 °C [59] using bromo–ester difunctionalized PXTPCLBr for 24 h in the presence of CuBr/PMDETA as the catalyst system with a mole ratio of [MMA or S]:[CuBr]:[PMDETA]:[PXTPCLBr] = 100:2:2:1 in toluene [80]. The resulting copolymers were characterized by FT-IR, ^1^H NMR, TGA, and GPC. As seen in NMR, the composition of the triblock copolymers remains richer in methacrylate and styrenic segments. The conversions of monomers were determined gravimetrically. The results and conditions of polymerization for each monomer are given in Table 2. FT-IR spectra of PCL-macroinitiated-block copolymers are shown in Figure 3e,f. Figure 3e shows the spectrum of PXT(PMMA-*b*-PCL-*b*-PMMA) triblock copolymers. The characteristic absorption peak of the carbonyl groups in PMMA and PCL segments was observed at around 1723 cm^−1^ with high relative intensity, which corresponds to the ester functionalities of these segments. The band at 1390–1590 cm^−1^ can be attributed to the combined vibrations of C–H bonds of the –CH_3_ groups in PMMA and C–H groups of the CH_2_ in PCL. The α-methyl groups’ vibrations were monitored at 1387 and 750 cm^−1^. In addition, stretching vibrations, which belong to C–O bonds, were observed in the region of 1140–1250 cm^−1^ for PMMA and PCL segments. C–H bond stretching vibrations of methyl and methylene groups’ stretching vibrations appeared at 2992 and 2950 cm^−1^, and are in good agreement with previous data reported in the literature [81,82].

The FT-IR spectrum (Figure 3f) allows the comparison of a polymer chemical structure with that of macroinitiator PCL (Figure 3d), as discussed above. In Figure 3f, PXT(PS-*b*-PCL-*b*-PS) shows the characteristic stretching bands at 1734 and 1601 cm^−1^, which correspond to the carbonyl (C=O) of the PCL segment and the double bonding of PS units. The peaks at 3077, 3056, and 3025 cm^−1^ assigned to aromatic C–H stretching vibrations and the peaks at 1451 and 1492 cm^−1^ due to C=C stretching vibrations were also observed as a result of polymerization. The absorption peaks at 752 and 696 cm^−1^ are attributed to C–H bending vibration, and those at 2921 and 2849 cm^−1^ to methylene stretching vibrations. In addition to these values, the presence of the overtones corresponding to PS segments between 1800 and 2000 cm^−1^ supports the formation of block copolymers [57]. 

^1^HNMR spectra of block copolymers are depicted in Figure 8e,f. For PXT(PMMA-*b*-PCL-*b*-PMMA) (in Figure 8e), the spectrum shows peaks at 3.59 ppm (O–C**H**_3_ in PMMA, H^m^), 1.11–0.82 ppm (–C**H**_3_ in PMMA H^k^), 2.11–1.71 ppm (–C**H**_2_ in PMMA H^j^), 2.36 ppm (H^k^), and 4.06 ppm (H^i^) methylene protons, –C**H**_2_O–, in PCL). For PXT(PS-*b*-PCL-*b*-PS), in Figure 8f, the peaks at 6.28–7.40 ppm, 4.06 ppm, and 2.36 ppm were attributed to the protons of aromatic (Ar–**H**, in PS, H^q+r^), (C**H**_2_–O, in PCL, H^i^), and (COO–C**H**_2_–, in PCL, H^e^), respectively. The overlapped peaks 2.0–0.9 ppm, which are attributed to protons of methylene (H^f+h+g^) of PCL and methine (H^p^) and methylene (H^n^) of PS, have a similar look as described above [83,84].

**Figure 9:**
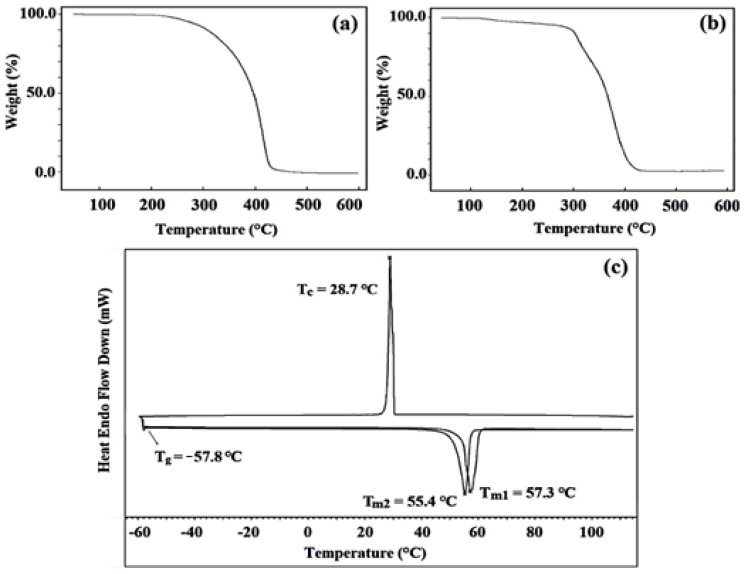
TGA curves of PXTPCLOH (**a**), PXT(PMMA-*b*-PCL-*b*-PMMA) (**b**), DSC trace of PXTPCLOH (**c**).

The thermal stability of PXTPCLOH and PXT(PMMA–*b*–PCL–*b*–PMMA) was evaluated by TGA at a rate of 20 °C min^−1^ between room temperature and 600 °C. In Figure 9a, the decomposition of PXTPCLOH starts at around 248 °C and completes at 444 °C. At higher temperatures, 271 °C, 95% of PCL was decomposed, with a maximum decomposition rate at 418 °C. As seen from the thermogram of (Figure 9b), PXT(PMMA-*b*-PCL-*b*-PMMA) shows two maximus rates, T_d1_ = 310.8 °C and T_d2_ = 377.7 °C.

The thermal stability of PCL was also evaluated by DSC, and the chromatogram showed unimodal melting peaks T_m1_ = 57.3 °C and T_m2_ = 55.4 °C, which denote the maximal melting temperatures of the polymer in the first and second heating runs, respectively. As can be seen in Figure 9c, PCL exhibited a crystallization peak at T_m1_ = 28.7 °C in the cooling run, and the *T*_g_ value of PCL was found to be -57.8 °C. These results agree with the values reported in the literature [85,86].

### 3.5. Measurement of the Heavy–Metal–Binding Properties of PXTPCLOH and PXTPS

The extraction abilities of PXTPCLOH and PXTPS were investigated under liquid–liquid phase conditions using heavy metal picrates (Ag^+^, Cd^2+^, Cu^2+^, Hg^2+^, and Pb^2+^) as substrates by measuring –the amounts of picrate in the 1,2-dichloroethane phase with UV-Vis.

As can be seen in Table 3, the highest extraction affinities of PXTPCLOH and PXTPS were determined to be 58.62 and 43.03% for Hg^2+^, respectively. The extraction affinities of PXTPCLOH for Ag^+^, Cd^2+^, Pb^2+^, Cu^2+^, and Zn^2+^ were determined to be 47.30, 42.86, 12.63, 32.04, and 21.32%, respectively. The extraction of affinities of PXTPS for Ag^+^, Cd^2+^, Pb^2+^, Cu^2+^, and Zn^2+^ were determined as 37.56, 29.35, 8.79, 17.25, and 14.64%, respectively. The higher extractability of PCL can be attributed to an increase in the number of oxygen donor atoms and terminal hydroxyl groups in PCL. In addition to these comments, it can be said that soft sulfur atoms in initiators (PXTOH or PXTBr) contribute to the extractability of each studied polymer. λ_max_ of metal picrates for Hg^2+^, Ag^+^, Cd^2+^, Pb^2+^, Cu^2+^, and Zn^2+^ were also determined as 352, 346, 351, 347, 348, and 352, respectively.

Joel et al. investigated the percent extractability of Cu^2+^, Pb^2+^, and Ag^+^ ions from an aqueous solution to the dichloromethane phase with 8H-DAB by using the liquid–liquid phase extraction method [87]. In this study, it was observed that the percent extractability of the Hg^2+^, Cd^2+^, and Cu^2+^ ions of PXTPCLOH and PXTPS polymers was higher compared to calix[4]arenediamine derivatives (7a’ and 7b) [88]. Moreover, the extractability of Ag^+^ ion by PXTPCLOH was higher than 8HDAB [87].

The higher extractability of PCL can be attributed to an increase in the number of oxygen donor atoms and terminal hydroxyl groups in PCL. In addition to these comments, it can be said that soft sulfur atoms in initiators (PXTOH or PXTBr) contribute to the extractability of each studied polymer. The lower extractability of PS for Cu^2+^ (when the extractabilities of polymers are compared, the one with the most difference in extraction) can be attributed to the possibility that the copper is used as a catalyst in ATRP and may bind to some extent. The higher values for Hg^2+^, Ag^+^, and Cd^2+^ were expected results. Therefore, sulfur–containing compounds are especially suitable for complexation with heavy metal ions such as Hg^2+^, Ag^+^, and Cd^2+^ due to the softness of sulfur [89].

## 4. Conclusions

In this work, the novel TBCs consisting of PS and PMMA were synthesized by two different pathways using novel ATRP (PXTBr) and ROP (PXTOH) initiators. We chose the sulfur-containing compound as a novel initiator because sulfur-containing initiators were relatively less commonly used than those containing oxygen and nitrogen. Moreover, sulfur–containing compounds can degrade at high temperatures, making them difficult to use in ATRP. In addition to the disadvantages mentioned above, we aimed to observe how polymerization proceeds with sulfur-containing initiators, while low efficiency was expected because copper(I) has a soft acid character and sulfur has a soft base character, allowing easy coordination with them.

We first used the sulfur-containing initiator in macroinitiator synthesis to investigate ATRP activity. The novel bifunctional ATRP initiator (PXTBr) was synthesized and employed for polymerization of MMA and S using CuBr/PMDETA as a catalyst system in bulk.

In addition to PMMA- and PS-based macroinitiators, di(α-bromoester) end-functionalized PCL-based PXTPCLBr was prepared by esterification of the hydroxyl end groups of PXTPCLOH synthesized by Sn(Oct)_2_-catalyzed ROP of *ε*-CL in bulk using the difunctional initiator ROP (PXTOH). ABA–type block copolymers were synthesized by second ATRP of MMA and S as monomers using novel (PMMA-, PS-, and PCL-based) macroinitiators and CuBr/PMDETA as catalyst systems in toluene or DMF at different temperatures. In the final step, the heavy metal extraction abilities of synthesized polymers (PXTPCL and PXTPS) were also investigated under liquid–liquid phase conditions using heavy metal picrates (Ag^+^, Cd^2+^, Cu^2+^, Hg^2+^, Pb^2+^, and Zn^2+^) as substrates and measuring with UV–Vis the amounts of picrate in the 1,2-dichloroethane phase before and after treatment with polymers. The extraction affinity of polymers for Hg^2+^ was found to be highest in the liquid–liquid phase extraction experiments. The structures of the products were elucidated using FT–IR, ESI–MS, ^1^H NMR, ^13^C NMR, GPC, TGA, DSC, and SEM methods. The M_n_ values of the macroinitiators increased with time for ATRP polymerizations of both MMA and S. As a result, it was observed that the synthesized polymers had a high molecular weight by GPC analysis and the desired PDI. PXT(PMMA-*b*-PCL-*b*-PMMA) showed two maximums at 310.8 °C and 377.7 °C. The surfaces of polymers are rough and porous. For example, PXT(PMMA-*b*-PS-*b*-PMMA) is rough, and macro and microporous.

We also investigated the extraction properties of PXTPCLOH and PXTPS, whose molar masses (GPC) are close to each other, under liquid–liquid phase conditions using heavy metal picrates (Ag^+^, Cd^2+^, Cu^2+^, Hg^2+^, and Pb^2+^) as substrates by measuring with UV-Vis the amounts of picrate in the 1,2-dichloroethane phase before and after treatment with polymers. The extraction of metal picrates from the aqueous phase to the 1,2-dichloroethane phase by the synthesized PCL and PS was examined by liquid–liquid extraction. The higher extractability of PCL can be attributed to an increase in the number of oxygen donor atoms and terminal hydroxyl groups in PCL. In addition to these comments, it can be said that soft sulfur atoms in initiators (PXTOH or PXTBr) contribute to the extractability of each studied polymer. The lower extractability of PS for Cu^2+^ (when the extractabilities of polymers are compared, the one with the most difference in extraction) can be attributed to the possibility that the copper is used as a catalyst in ATRP and may bind to some extent. Higher values for Hg^2+^, Ag^+^, and Cd^2+^ were expected results. Therefore, sulfur–containing compounds are especially suitable for complexation with heavy metal ions such as Hg^2+^, Ag^+^, and Cd^2+^ due to the softness of sulfur.

## Figures and Tables

**Table 1:** Results and conditions of the polymerization of MMA and S with the initiator (PXTBr) in bulk by ATRP.

Entry	Time (h)	Conv ^c^ (%)	M_n,th_^d^	M_n,GPC_^e^	*Đ* ^e^	*f* ^f^
PM-1 ^a^	0.5	21.4	2696	6500	1.28	0.41
PM-2 ^a^	1.0	55.3	6086	17,440	1.31	0.35
PM-3 ^a^	2.0	65.1	7066	20,300	1.75	0.35
PS-1 ^b^	0.5	22.8	2927	6780	1.30	0.43
PS-2 ^b^	1.0	25.7	3228	8700	1.34	0.37
PS-3 ^b^	2.0	80.3	8907	25,270	1.49	0.35

^a^ Polymerization conditions: [MMA]_0_:[CuBr]_0_:[PMDETA]_0_:[I]_0_ = 50:1:1:0.5 polymerization temperature = 90 °C. ^b^ Polymerization conditions: [S]_0_:[CuBr]_0_:[PMDETA]_0_:[I]_0_ = 50:1:1:0.5 polymerization temperature = 110 °C. ^c^ $Conversion\%=\frac{m_{yield}-m_{macroinitiator}}{m_{monomer}}\times 100$. ^d^ M_n,th_ of PMMA or PS. M_n,th_ = [M]/[I] × M_monomer_ × Conversion(%) + M_initiator_ (M_MMA_ = 100.12, M_S_ = 104.15 and M_initiator_ = 553.98 g/mol). ^e^ M_n,GPC_ and *Đ* determined by GPC analysis with PMMA standards. THF was used as eluent. The ^f^ of each experimental run was calculated from the equation (*f*) = M_n,th_/M_n,GPC_.

**Table 2:** Results and conditions of the block copolymers by ATRP.

Entry	Temp (°C)	M_n,th_ × 10^4^	^f^ M_n,GPC_ × 10^4^	*Đ* ^f^
ABA–1 ^a^	110	3.41	4.60	1.52
ABA–2 ^b^	100	3.00	3.12	1.34
ABA–3 ^c^	110	1.85	2.23	1.41
ABA–4 ^d^	100	1.81	2.16	1.31
ABA–5 ^e^	100	7.19	13.15	1.21
ABA–6 ^e^	100	7.65	8.28	1.40

^a^ Polymerization conditions: [S]_0_:[CuBr]_0_:[PMDETA]_0_:[PM-1]_0_ = 1000:2:2:1 in DMF. ^b^ Polymerization conditions:[MMA]_0_:[CuBr]_0_:[PMDETA]_0_:[PS-1]_0_ = 1000:2:2:1 in DMF. ^c^ Polymerization conditions: [S]_0_:[CuBr]_0_:[PMDETA]_0_:[PM-1]_0_ = 500:2:2:1 in DMF. ^d^ Polymerization conditions:[MMA]_0_:– [CuBr]_0_:[PMDETA]_0_:[PS-1]_0_ = 500:2:2:1 in DMF. ^e^ Polymerization conditions:[S or MMA]_0_:[CuBr]_0_:–[PMDETA]_0_:[PXTPCLBr]_0_ = 1000:2:2:1 in toluene. ^f^ M_n,GPC_ and *Đ* determined by GPC analysis with PMMA standards. THF was used as eluent.

**Table 3:** Heavy metal picrate extractions for PXTPCLOH or PXTPS under liquid–liquid phase conditions.

	Extractability (%)					
Metal ion	Hg^2+^	Ag^+^	Cd^2+^	Pb^2+^	Cu^2+^	Zn^2+^
PXTPCLOH	58.62	47.30	42.86	12.63	32.04	21.32
PXTPS	43.03	37.56	29.35	8.79	17.25	14.64
8H–DAB [87]	–	42.36		35.22	35.32	–
Calix[4]arenediamine derivative(7a’) [88]	46.61	–	33.06	–	14.22	–
Calix[4]arenediamine derivative(7b) [88]	19.07	–	8.26	–	9.91	–

## Data Availability

All data and materials produced from this study are public accessible.
